# Vitamin D Deficiency in Early Pregnancy

**DOI:** 10.1371/journal.pone.0123763

**Published:** 2015-04-21

**Authors:** Shannon K. Flood-Nichols, Deborah Tinnemore, Raywin R. Huang, Peter G. Napolitano, Danielle L. Ippolito

**Affiliations:** 1 Division of Maternal-Fetal Medicine, Madigan Army Medical Center, Joint Base Lewis-McChord, Tacoma, Washington, United States of America; 2 Department of Clinical Investigation, Madigan Army Medical Center, Joint Base Lewis-McChord, Tacoma, Washington, United States of America; Medical Faculty, Otto-von-Guericke University Magdeburg, GERMANY

## Abstract

**Objective:**

Vitamin D deficiency is a common problem in reproductive-aged women in the United States. The effect of vitamin D deficiency in pregnancy is unknown, but has been associated with adverse pregnancy outcomes. The objective of this study was to analyze the relationship between vitamin D deficiency in the first trimester and subsequent clinical outcomes.

**Study Design:**

This is a retrospective cohort study. Plasma was collected in the first trimester from 310 nulliparous women with singleton gestations without significant medical problems. Competitive enzymatic vitamin D assays were performed on banked plasma specimens and pregnancy outcomes were collected after delivery. Logistic regression was performed on patients stratified by plasma vitamin D concentration and the following combined clinical outcomes: preeclampsia, preterm delivery, intrauterine growth restriction, gestational diabetes, and spontaneous abortion.

**Results:**

Vitamin D concentrations were obtained from 235 patients (mean age 24.3 years, range 18-40 years). Seventy percent of our study population was vitamin D insufficient with a serum concentration less than 30 ng/mL (mean serum concentration 27.6 ng/mL, range 13-71.6 ng/mL). Logistic regression was performed adjusting for age, race, body mass index, tobacco use, and time of year. Adverse pregnancy outcomes included preeclampsia, growth restriction, preterm delivery, gestational diabetes, and spontaneous abortion. There was no association between vitamin D deficiency and composite adverse pregnancy outcomes with an adjusted odds ratio of 1.01 (*p* value 0.738, 95% confidence intervals 0.961-1.057).

**Conclusion:**

Vitamin D deficiency did not associate with adverse pregnancy outcomes in this study population. However, the high percentage of affected individuals highlights the prevalence of vitamin D deficiency in young, reproductive-aged women.

## Introduction

Vitamin D deficiency is a common problem in reproductive aged women in industrialized countries and its prevalence may be increasing [1]. The etiology for this increase is likely multifactorial, but due in part to low dietary intake of vitamin D and limited exposure to sunlight. Certain high risk groups for vitamin D deficiency in pregnancy have been identified, including vegetarians, women with limited sun exposure (e.g., those who live in cold climates, northern latitudes, or wear sun and winter protective clothing), and ethnic minorities, especially those with darker skin [2]. Vitamin D deficiency is also more common among heavier women than leaner individuals [3]. During pregnancy, severe maternal vitamin D deficiency, as defined as serum 25-hydroxyvitamin D (25-OH vitamin D) concentrations less than 5 ng/mL, has been associated with disordered skeletal homeostasis, congenital rickets, and fractures of the newborn [4]. However, the effect of less severe vitamin D deficiency and insufficiency on maternal and fetal outcomes during pregnancy is less clear. Recent observational and randomized control trials have attempted to investigate this issue, but there remains limited guidance on the management of vitamin D deficiency during pregnancy.

Vitamin D deficiency and insufficiency have been associated with a variety of adverse maternal and fetal outcomes, ranging from preeclampsia, gestational diabetes, preterm delivery, intrauterine growth restriction, spontaneous abortion, and cesarean section [5–10]. However, other studies have demonstrated no association between vitamin D status and adverse pregnancy outcomes [11–12]. The majority of randomized control trials and observational studies measure vitamin D late in the pregnancy, when prenatal vitamins containing 400 IU of vitamin D have been prescribed [13]. Thus, we sought to determine if vitamin D deficiency in the first trimester of nulliparous patients is associated with clinical outcomes most associated with significant maternal and fetal morbidity and mortality: preeclampsia, gestational diabetes, preterm delivery, spontaneous abortion, and growth restriction. Nulliparous women were studied to decrease the effect of prior pregnancy complications on study outcomes. The objective of this study was to analyze the relationship between hypovitaminosis D in the first trimester and subsequent clinical outcomes in a population of nulliparous women.

## Study Design

### Participants

This study was approved by the Madigan Army Medical Center Institutional Review Board (Protocol #205031). Investigators adhered to the institutional policies for protection of human participants. All participants provided informed consent. Healthy, nulliparous women aged 18 years or older without a history of chronic medical conditions (e.g., preexisting diabetes mellitus (type 1 or 2), neurological disorders, cardiovascular anomalies, etc.) or infertility treatment were approached to participate in the study during their initial intake appointment at 8 to 12 weeks gestation by first day of the last menstrual period. All gestational ages were confirmed with either a first or a second trimester ultrasound, verified by chart review at study completion. Patients with predictors for hypovitaminosis D, such as anticonvulsant use, renal and cardiovascular disease, preexisting diabetes mellitus (type 1 or 2), were not eligible for recruitment at the beginning of the study. Women with a prior pregnancy that had progressed beyond the first trimester and resulted in a fetal loss or intrauterine fetal demise were also not approached for study enrollment. All patients were recruited from Madigan Army Medical Center, a tertiary military medical center with 2279 deliveries in 2014. All patients included in this study received their prenatal care and delivered at Madigan. Vitals including body mass index (BMI) and blood pressure were measured at initial and all subsequent obstetric appointments in accordance with standard-of-care procedures. Additional demographic data was obtained including patient age, race, and tobacco use during pregnancy. Information extracted from delivery records included vital signs and gestational age at time of admission for delivery.

Based on clinical outcomes data, patients were segregated into cohorts of uncomplicated and complicated pregnancies. Complicated pregnancies were defined by the presence of adverse pregnancy outcomes including preeclampsia, gestational diabetes, preterm delivery, growth restriction, and spontaneous abortion. Preeclampsia and gestational diabetes were diagnosed as described in the American College of Obstetricians and Gynecologists practice bulletins [14–15]. Preterm delivery was considered as a spontaneous preterm birth less than 37 weeks gestational age due to preterm labor or preterm premature rupture of membranes and growth restriction was defined by estimated fetal weight less than the 10^th^ percentile for gestational age. Spontaneous abortion was defined as spontaneous abortion in the first trimester after enrollment in the study [16–17].

### Plasma preparation and vitamin D ELISA

Maternal blood was collected by venipuncture in vacutainer EDTA-containing tubes at 5 to 12 weeks gestational age and the date of the blood draw was recorded. Plasma was separated by centrifugation at 1500x*g* at 4°C in a Sorvall RC3C centrifuge (Sorvall Instruments, ThermoFisher Scientific, West Palm Beach, FL). Plasma was supplemented with protease inhibitors (Roche Diagnostics (1 836 145), Mannheim, Germany). Samples were aliquoted into multiple tubes and stored at -140°C.Total 25-OH vitamin D concentrations (ng/mL) were quantified in patient plasma samples by competitive enzymatic linked immunosorption assay (Catalog #DZ688A, Diazyme Laboratories, Poway, CA). Researchers were blinded to the pregnancy outcomes while performing the assays. Briefly, 25-OH vitamin D was dissociated from its serum transporter by acetonitrile extraction. Serum 25-OH vitamin D activated the enzyme beta-galactosidase and its substrate chlorophenol red-beta-D galactopyranoside, producing a colorimetric reaction product (chlorophenol red) detectable at 560nm by microplate reader (Synergy HT, Biotek, Winooski, VA). The amount of chlorophenol red was directly proportional to serum 25-OH vitamin D concentration. Concentrations were determined by interpolating to a standard curve generated by plotting absorbencies of calibrators at the following concentrations: 0, 14, 25, 45, 65, and 95 ng/ml (Diazyme #DZ688A-CAL). Assay quality control was assessed by measuring 25-OH vitamin D concentrations of assay controls run in parallel with patient samples. Assay fidelity was confirmed by liquid chromatography and tandem mass spectrometry as per the manufacturer’s standard quality control procedures (Diazyme). Assay controls were 2 factory-supplied serum specimens containing approximately 24.4 ng/ml and 43.4 ng/ml 25-OH vitamin D (#DZ688A-CON, Diazyme; exact concentrations were lot-specific) as measured by an alternative ELISA strategy (Immunodiagnostic Systems, Scottsdale, AZ). Only assays in which controls were accurately measured from calibrator standard curves were included in the final data analysis. A quantitative comparative analysis of 29 standards (range of 20-113ng/ml) was undertaken by both liquid-chromatography/mass spectrometry (LC/MS) and the Diazyme immunoassay to determine the fidelity of the immunoassay relative to mass spectrometry.

The ELISA assay had an interassay variability of 9.3% and intraassay variability of 7.8%. Plasma vitamin D Diazyme vitamin D immunoassay performed comparably to LC/MS mass spectrometry. The comparative analysis resulted in a correlation coefficient of 0.95 between the two methods (-8.23% bias).

### Categorization of vitamin D status

Recently published criteria from the Institute of Medicine were used to categorize vitamin D status by plasma 25-OH vitamin D concentrations: severe deficiency (<10 ng/ml [25nmol/L]); deficiency (< 20 ng/ml [50nmol/L]); insufficiency (21–29 ng/ml [51–74 nmol/L]); and sufficiency (≥ 30 ng/ml [75nmol/L] [18].

### Statistics

We estimated the approximate number of patients needed for adequate statistical power with an expected complication rate of 25% among all vitamin D categories. Assuming an α of 0.05, with statistical power of 80%, an effect size of 1.33 would require a sample size of at least 20 patients per category (sufficient, >30 ng/ml; insufficient, 21–29 ng/ml; or deficient, <20 ng/ml). However, we enrolled a total of 310 patients to account for possible attrition. Multivariable logistic regression was performed using Statistical Package for Social Sciences (SPSS) version 19. Chi-square analysis was performed on categorical variables and analysis of variance was performed on continuous variables. Chi square analysis was performed on the categorical variables (race, BMI at time of enrollment and plasma sampling, season, tobacco use, duty status, delivery mode) and analysis of variance (ANOVA) was performed on the continuous variables (maternal age and gestational age at intake). For logistic regression analysis, dependent variables were pregnancy clinical outcomes categorized as follows: no complications, preeclampsia, preterm delivery, intrauterine growth restriction, gestational diabetes, and spontaneous abortion. Independent variables were vitamin D category according to criteria recently published by the Institute of Medicine and Endocrine Society (i.e., sufficient, >30 ng/ml; insufficient, 21–29 ng/ml; or deficient, <21 ng/ml) [18–19]. Both unadjusted odds ratios and odds ratios adjusted for BMI, season, tobacco use, and ethnicity were calculated. Statistical significance was selected to be 0.05.

## Results

Of the 315 patients approached for study participation, a total of 310 patients agreed to participate and gave informed consent according to the Institutional Review Board approval of the study protocol (response rate: 98.7%). A total of 235 patients were included in the final analysis (mean age 24.3 years, range 18–40 years). Of the original 310 patients recruited, 75 were not included in the study because they withdrew from the study (n = 8), did not deliver at our institution (n = 44), underwent elective pregnancy termination (n = 3), were lost to follow-up (n = 18) or were diagnosed with twin gestations after enrollment (n = 2) (attrition rate of 24.2%). Of the 235 patients included in the study, 41 of them had a prior pregnancy which had resulted in a spontaneous first trimester miscarriage or elective termination. Mean gestational age of recruitment and vitamin D plasma measurement was 62.2 ± 29.7 days (8.9 ± 4.2 weeks).A pregnancy was considered complicated if the patient was diagnosed with any of the following adverse pregnancy outcomes: preeclampsia, gestational diabetes, spontaneous preterm delivery, intrauterine growth restriction, or spontaneous abortion (Table 1). For the purposes of this study, patients who did not experience one of these complications were categorized as uncomplicated. Approximately 25% of patients had complicated pregnancies. Frequencies of pregnancy complications are tabulated in Table 1 and compared with predicted US and institutional frequencies for preeclampsia [20–26], preterm delivery [27–28],intrauterine growth restriction [29–30], gestational diabetes mellitus [31–36], and spontaneous abortion [37–38]. As shown in Table 1, three patients categorized in the spontaneous preterm delivery category had comorbidities including intrauterine growth restriction (IUGR) and gestational diabetes. These patients were not included in the IUGR or gestational diabetes categories for subsequent statistical analysis. Similarly, the 5 patients with a medically indicated preterm delivery subsequent to preeclampsia were not included in the preterm delivery categories for subsequent analysis. The predicted adverse event frequencies were comparable to national and international epidemiological predictions (Table 1). The patient population comprised active duty military or dependent spouses (Table 2). Information on patient time in the area prior to pregnancy, dietary intake of vitamin D, and sun exposure was not available. All patients were prescribed prenatal vitamins (approximately 400 IU/day) at their initial intake appointment, but compliance rates on use were unknown. When actual plasma vitamin D concentrations were determined for the 235 patients included in the final analysis, technical reproducibility among replicates was assessed by calculating the coefficient of variation (CV) among calibrators, controls, and samples. CVs were 15.8% for calibrators, 8.5% for assay controls, and 7.8% for patient plasma samples. Ten percent of our study population was vitamin D deficient with a serum concentration less than 20 ng/mL (50 nmol/L); 60% were vitamin D insufficient (serum concentrations between 20–29 ng/mL or 50–74 nmol/L); and 30% of our study population were vitamin D sufficient with serum concentrations above 30 ng/mL (75 nmol/L) (Table 2). None of our patients were classifiable as severely vitamin D deficient (<10 ng/ml or 25 nmol/L). The mean serum 25-OH vitamin D concentration for our study population was 27.6 ng/mL (range 13–71.6 ng/mL).

**Table 1:** Frequency of clinical outcomes compared to national and global epidemiology.

	Pregnancy Outcome n (% of 235 total)	Comorbidities	Predicted Population US (%)	Frequencies Global (%)	Madigan 2014 (%)
**None**	176 (75%)	--	--	--	--
**Preeclampsia**	19 (8%)	5 preterm deliveries	3–10% [22,24–26]	2–17% [20, 23]	3%
**Spontaneous preterm delivery**	10 (4%)	2 IUGR, 1 GDM	11.3% [27]	9.6% [28]	9%
**IUGR**	9 (4%)	--	8.6% [29]	3–7% [30]	0.6%
**GDM**	5 (2%)	--	4.6–9.2% [33]	4.1–27.5% [36]	4%
**Spontaneous abortion**	16 (7%)	--	8–20% [37,38]		6%

IUGR, intrauterine growth restriction; GDM, gestational diabetes mellitus.

**Table 2:** Demographics of the study population by vitamin D classification.

	Total	Vitamin D Sufficient (>30 ng/ml)	Vitamin D Insufficient (21–29 ng/ml)	Vitamin D Deficient (<20 ng/ml)	p value**
	n = 235 (%)	n = 70 (30%)	n = 141 (60%)	n = 24 (10%)	
**Age (years)** *	24.3 ± 4.4	24.5 ± 4.2	24.6 ± 4.5	22.5 ± 3.7	0.08
**Gestational Age at Analysis (days)** *	62.2 ± 29.7	61.8 ± 13.7	62.2 ± 36.9	63.0 ± 11.2	0.99
	**n (% of 235)**	**n (% of 70)**	**n (% of 141)**	**n (% of 24)**	**p value** **
**Race**					0.03
**Caucasian**	183 (77.9)	57 (81.4)	114 (80.9)	12 (50)	
**African American**	16 (6.8)	3 (4.3)	10 (7.1)	3 (12.5)	
**Asian**	6 (2.6)	1 (1.4)	3 (2.1)	2 (8.3)	
**Other**	30 (12.8)	9 (12.9)	14 (9.9)	7 (29.2)	
**Body Mass Index**					0.36
**<25**	111 (47.2)	39 (55.7)	62 (44)	10 (41.7)	
**25–30**	82 (34.9)	21 (30.0)	50 (35.5)	11 (45.8)	
**>30**	42 (17.9)	10 (14.3)	29 (20.5)	3 (12.5)	
**Season**					0.22
**Winter**	89 (37.9)	22 (31.4)	61 (43.3)	6 (25.0)	
**Spring**	45 (19.1)	16 (22.9)	25 (17.7)	4 (16.6)	
**Summer**	53 (22.6)	20 (28.6)	26 (18.4)	7 (29.2)	
**Fall**	48 (20.4)	12 (17.1)	29 (20.6)	7 (29.2)	
**Mode of Delivery**					0.41
**Vaginal**	155 (66.0)	47 (67.1)	95 (67.4)	13 (54.2)	
**Cesarean section**	63 (26.8)	18 (25.7)	36 (25.5)	9 (37.5)	
**IUFD or SAB**	17 (7.2)	5 (7.1)	10 (7.1)	2 (8.3)	
**Duty Status**					0.12
**Active Duty**	83 (35.3)	22 (31.4)	48 (34.0)	13 (54.2)	
**Dependent**	152 (64.6)	48 (68.6)	93 (66.0)	11 (45.8)	
**Tobacco Use**					0.67
**Yes**	15 (6.4)	3 (4.3)	10 (7.1)	2 (8.3)	
**No**	220 (93.6)	67 (95.7)	131 (92.9)	22 (91.7)	

*Expressed in mean ± standard deviation

**, p value calculated by analysis of variance for continuous variables and chi-square analysis for categorical variables

IUFD, intrauterine fetal demise; SAB, spontaneous abortion.

Forty-seven percent of our study population was of normal weight with a BMI <25 (Table 2). Thirty-five percent were overweight with a BMI at intake between 25–30, and about 18% of our patients were obese with a BMI >30. Most of our study population was Caucasian (78%), with 7% African American, 3% Asian, and 12% other, unspecified races. Thirty-eight percent of the blood draws occurred in the winter, 19% in the spring, 22% in the summer, and 21% in the fall. About 6% of our study population used tobacco during pregnancy. Statistical associations between vitamin D categorization and demographic variables are depicted in Table 2. Race was the only statistically different variable among the vitamin D groups, with significantly more African American, Asian, and other unspecified racial groups in the vitamin D deficient category compared to the insufficient and sufficient groups (p = 0.03).

The distribution of clinical outcomes among the vitamin D categories is depicted in Table 3. Study population percentages and with medians and ranges of vitamin D measured in plasma were calculated. Preeclampsia was sub-stratified into severe, early onset preeclampsia and mild, late-onset preeclampsia.

**Table 3:** Plasma vitamin D concentrations in women according to clinical outcome.

	n (% of 235 total)	median vitamin D concentration in ng/ml (range)
**No complications**	176 (75)	27.4 (13.0–71.6)
**All complications**	59 (25)	26.5 (13.2–42.3)
**Preeclampsia**	19 (8)	26.5 (16.1–35.9)
**Severe, early onset**	5 (2)	27.1 (18.0–33.8)
**Mild, late onset**	14 (6)	27.7 (16.1–35.9)
**Spontaneous Preterm delivery**	10 (4)	28.4 (19.9–42.3)
**IUGR**	9 (4)	27.0 (16.6–38.8)
**GDM**	5 (2)	22.2 (20.5–31.9)
**Spontaneous Abortion**	16 (7)	25.1 (13.2–38.8)

IUGR, intrauterine growth restriction; GDM, gestational diabetes mellitus.

Plasma vitamin D concentrations were subdivided into 3 categories based on the Institution of Medicine and Endocrine Society criteria for vitamin D categorization: sufficient (>30ng/ml [75nmol/L]), insufficient (21-29ng/ml [50-75nmol/L], or deficient [<50nmol/L). None of our patients were categorized as severely deficient (<10ng/ml [25nmol/L]) (Table 4). Because there were too few patient in the category of vitamin D deficient (<20ng/ml [50nmol/L]) for meaningful statistical analysis, patients categorized as either vitamin D insufficient or deficient were combined into one category (insufficient/deficient, <30ng/ml [75nmol/L]) for subsequent statistical analysis (Table 5).

**Table 4:** Distribution of clinical outcomes in women categorized as Vitamin D deficient, insufficient, or sufficient.

Vitamin D Category	No Complications n (% of 176)	All Complications n (% of 59)	Preeclampsia n (% of 19)	Spontaneous Preterm delivery n (% of 10)	IUGR n (% of 9)	GDM n (% of 5)	Spontaneous Fetal loss n (% of 16)
**Deficient (<20 ng/ml)**	18 (10)	6 (10)	2 (11)	1 (10)	1 (11)	0 (0)	2 (13)
**(n = 24)**							
**Insufficient (21–29 ng/ml)**	105 (60)	36 (61)	10 (53)	6 (60)	7 (78)	3 (60)	10 (63)
**(n = 141)**							
**Sufficient (>30 ng/ml)**	53 (30)	17 (29)	7 (37)	3 (30)	1 (11)	2 (40)	4 (25)
**(n = 70)**							

IUGR, intrauterine growth restriction; GDM, gestational diabetes mellitus.

**Table 5:** Logistic regression analysis of clinical outcomes in women categorized as vitamin D sufficient compared to vitamin D insufficient or deficient women.

	Vitamin D Sufficient (≥30 ng/ml)	Vitamin D Insufficient or Deficient (<30 ng/ml)	
	n (% of 70)	n (% of 165)	Adjusted** OR (95% CI)*
**No complications**	53 (75.7)	123 (74.5)	-
**All complications**	17 (24.2)	42 (25.5)	1.01 (0.947–1.164)
**Preeclampsia**	7 (10)	12 (7.2)	1.36 (0.48–3.88)
**Spontaneous Preterm delivery**	3 (4.3)	7 (4.2)	0.78 (0.17–3.55)
**IUGR**	1 (1.4)	8 (4.8)	0.33 (0.38–2.83)
**GDM**	2 (2.9)	3 (1.8)	2.60 (0.28–27.24)
**Spontaneous Abortion**	4 (5.7)	12 (7.2)	0.65 (0.18–2.28)

*, note: none of the differences were p<0.05 by logistic regression (chi-square analysis)

**, Adjusted for BMI, season, ethnicity, and tobacco use

OR, odds ratio

IUGR, intrauterine growth restriction; GDM, gestational diabetes mellitus.

There was no association between vitamin D deficiency in the first trimester and adverse pregnancy outcomes with an adjusted odds ratio of 1.01 (*p* value 0.738, 95% CI 0.947–1.164). Logistic regression was performed on combined outcomes and individual outcomes separately. There was no association between first trimester vitamin D levels and subsequent development of combined complications, or complications sub-stratified into preeclampsia, gestational diabetes, preterm delivery, intrauterine growth restriction (IUGR), or spontaneous abortion when each outcome was evaluated individually (Table 5). Odds ratios were adjusted for BMI, ethnicity, tobacco use, and season. There was no association between first trimester vitamin D levels and subsequent development of composite adverse pregnancy complications (including preeclampsia [severe, early onset or mild, late onset], gestational diabetes, preterm delivery, growth restriction, or spontaneous abortion (combined outcomes; p > 0.05 in Table 5).

## Discussion

**Fig 1:**
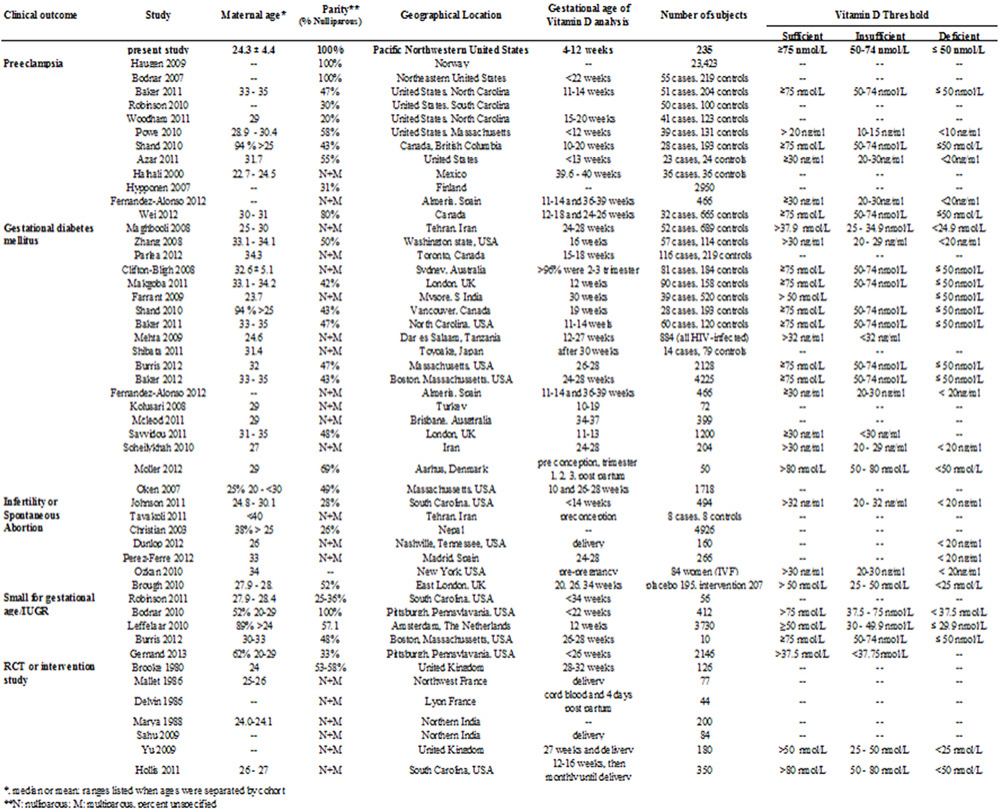
Comparison of study parameters in recent vitamin D observational and intervention studies.

This study investigated the prevalence of hypovitaminosis D in young (24.3 ± 4.4 years), nulliparous women at a tertiary military medical facility in the Pacific Northwestern United States at an average gestational age of 8.9 ± 4.2 weeks. Our study is distinctive as we examined a healthy population of women who should be at relatively lower risk for pregnancy complications. These women did not have preexisting hypertension, diabetes, or other medical problems that would increase their risk for pregnancy complications or vitamin D deficiency. Although we recruited only young, nulliparous, previously healthy women, the majority of our patients were either vitamin D deficient (10%, <20 ng/ml) or vitamin D insufficient (60%, 20–29 ng/ml) according to the criteria published by the Institute for Medicine.^18^ Of the 235 patients included in the study, 41 of them had a prior pregnancy which had resulted in a spontaneous first trimester miscarriage or elective termination. As these prior losses were not associated with a diagnosis of infertility, recurrent pregnancy loss or other medical conditions, we felt there would be minimal confounding by including them in our study. We found no association between hypovitaminosis D (plasma levels of <30 ng/ml) and the combined clinical outcomes of preeclampsia, preterm delivery, intrauterine growth restriction, gestational diabetes, or spontaneous abortion in this patient population. This study was not powered to detect associations between vitamin D status and these individual pregnancy complications. Fig 1 compares our study population with recently published cohort studies and randomized controlled trials (reviewed in Christesen [2012] and Wei [2013]) [13, 39].

Risk factors for vitamin D deficiency include inadequate sun or UV light exposure, physiologic factors (such as dark skin pigmentation, obesity, pregnancy, certain medical problems), and low vitamin D intake [2–3]. The prevalence of vitamin D deficiency in reproductive aged women appears to be increasing worldwide over the past two decades.^1,3,13^ Recent studies indicate the prevalence of vitamin D insufficiency and deficiency in nulliparous women, a result corroborated by our study [40]. The etiology of the decline of vitamin D status in women is likely multifactorial, but is due in part to increasing BMI and increased sun protection, coupled with decreased dietary intake of vitamin D, such as milk and other dairy products. Unfortunately, the increasing use of multivitamins and mineral dietary supplements in younger to older adults does not appear to be associated with a corresponding increase in serum 25-OH vitamin D concentrations [3, 19, 41]. Recent clinical guidelines suggest that doses of vitamin D greater than 600 IU/day may be needed to maintain adequate plasma levels greater than 30 ng/ml (75 nmol/L), but the appropriate standard is still debated [19, 42–43]. In recent randomized controlled studies, loading doses as high as 1000–4000 IU/day were needed before adequate vitamin D plasma concentrations were measured [18–19, 44].

Other risk factors for vitamin D deficiency include ethnicity, as darker skinned individuals have decreased vitamin D3 synthesis in response to ultraviolet light. Vitamin D deficiency has been proposed as a possible biologic explanation for racial disparities noted in adverse pregnancy outcomes including preeclampsia, spontaneous preterm birth, gestational diabetes and growth restriction [5]. Because vitamin D deficiency is so widespread, determining whether vitamin D deficiency is associated with adverse pregnancy outcomes is an area of major public health concern. It is unclear whether vitamin D deficiency is causal in the disparities in adverse pregnancy outcomes between Caucasians and African Americans. Out of 235 patients in our study, only about 7% were African American and thus we were not powered enough to examine this significance. However, even after adjusting for ethnicity in our analysis, there was still no significant difference between hypovitaminosis D and clinical outcome.

There are two forms of vitamin D in the body: vitamin D2 and vitamin D3. Measures of blood levels reflect both dietary intake as well as synthesis from exposure to the sun. The liver converts both forms of vitamin D to 25-OH vitamin D, which is the specific metabolite measured to determine vitamin D status. These levels are considered to be an accurate representation of the vitamin D status of an individual [1, 45]. A serum level of at least 20 ng/mL (50 nmol/L) is needed to avoid bone problems, and the optimal level for promoting health was recently published as 30 ng/mL (75 nmol/L) [2, 5, 18–19]. However, it is important to note that these thresholds were derived from a population of nonpregnant individuals and an optimal serum level during pregnancy has not been established.

Season and latitude dramatically alter vitamin D3 production [46–47]. In wintertime, the angle of the sun is so oblique that latitudes greater than 35 degrees receive almost no ultraviolet rays capable of stimulating vitamin D3 synthesis. As our study center is around 47 degrees latitude and 38% of our first trimester blood draws occurred in the winter, this certainly could contribute to the prevalence of vitamin D deficiency in our population. In addition, the Pacific Northwest experiences fewer sunny days than the rest of the country, with only 46% of daylight hours that are predominantly sunny or partly cloudy. For example, the greater Seattle area experiences an average of 8 and a half hours of daylight hours during December but only roughly 2 hours during the day are considered “sunny”. Compare this to South Carolina, which experiences over 6 hours of sunshine out of almost 10 hours of daylight during the same month. Unfortunately, data on individual daily sun exposure, travel and dietary intake are not available [48–49]. Other limitations and sources of possible bias for this study include low generalizability (small cohort of patients in the Pacific Northwestern United States), small sample size, self-selection of study subjects, and the high loss-to-follow-up rate in our study population. Information on the job description of active duty women was unavailable for this study, as was data on physical activity and location of duty station influencing sun exposure (i.e., indoors vs. outdoors). Body mass index in this population may not necessarily be indicative of overweight or obese given that some active duty women may have more muscle mass contributing to BMI measurement.

Several recent randomized controlled trials and observational cohort studies investigate a possible association between hypovitaminosis D and adverse clinical outcome in pregnancy (reviewed in Christesen [2012]) [13]. The role vitamin D plays in adverse clinical outcomes such as preeclampsia, gestational diabetes, preterm labor, growth restriction, and spontaneous abortion is speculative, but may involve vitamin D’s regulation of placental trophoblast invasion and angiogenesis, in addition to its anti-inflammatory properties [5]. For example, there is some evidence vitamin D affects transcription and function of genes responsible for trophoblast invasion and angiogenesis, two factors critical for placenta development. Vitamin D deficiency may predispose individuals to abnormal trophoblast invasion, reduced placental perfusion, and the subsequent cascade of events resulting in preeclampsia. Maternal vitamin D deficiency may also predispose women to an increased inflammatory response that characterizes preeclampsia, especially severe, early onset disease (Reviewed in Christesen [2012] and Wei [2013]) [13, 39]. In our cohort of young, nulliparous patients, we had a preeclampsia rate of 8%. However, vitamin D deficiency did not increase the odds of preeclampsia, even when stratifying by disease severity. This discrepancy may be explained in part by differences in our study population relative to other published cohort studies, including difference in parity, maternal age at intake, and/or gestational age of specimen collection (Fig 1) [6, 10–11, 40, 50–70]. During pregnancy, 25-OH vitamin D diffuses across the placenta and the fetus relies entirely on the vitamin D stores of the mother: if the mother is deficient, so is the fetus [5]. Thus, in combination with its immunomodulatory and anti-inflammatory properties, vitamin D may play a potential role in the prevention of preterm birth and small-for-gestational-age neonates. Vitamin D deficiency also has been shown to increase insulin resistance and reduce insulin secretion, which has shown to be a risk factor for gestational diabetes [5, 71–72]. However, whether vitamin D deficiency is a risk factor for gestational diabetes in itself or if vitamin D supplementation can prevent GDM is yet unknown [72].

Although vitamin D has a biologically possible role in all the aforementioned disorders, this was not demonstrated in our population as vitamin D deficiency did not increase the odds for adverse pregnancy outcome in our study. Study-specific parameters such as maternal demographics and parity may account for some of these differences in our cohort relative to published studies (Table 2 and Fig 1). For example, we had a lower incidence of gestational diabetes in our study population compared to what is reported in the literature [31–35, 73–75].Risk factors for gestational diabetes include women older than 25, obesity (BMI >30), history of a large baby or previously affected pregnancy, and women who are African American, American Indian, Asian American, Hispanic, Latina, or Pacific Islander [14]. We feel our low incidence of GDM is likely due to the young age (average age 24 years old), nulliparity, relatively low rate of obesity (17.9%), and predominantly white ethnicity (77%) of our study population. Obesity is also a known risk factor for vitamin D deficiency[73]. We reviewed the BMI of our study participants to see if there was any association between vitamin D deficiency classification and BMI. Out of the 235 patients analyzed, 42 (17.9%) had a prepregnancy BMI >30 and were considered obese. However, there was no significant difference in the distribution of these patients among the various vitamin D categories, with 10/70 (14.3%) women who were vitamin D sufficient, 29/141 (20.5%) vitamin D insufficient, and 3/24 (12.5%) vitamin D deficient (p = 0.22) (Table 2).

Our nulliparous patient population is also at different risk for certain pregnancy complications, such as preeclampsia and preterm delivery, compared to their multiparous counterparts. The prevalence of preeclampsia in the United States is about 3.4 percent, but 1.5-fold to 2-fold higher in first pregnancies [22]. In fact, most cases of preeclampsia occur in nulliparous women without significant medical history or risks factors [15]. Our nulliparous population may also be at lower risk for preterm delivery as one of the strongest clinical risk factors for preterm birth is a prior preterm birth [76]. Behavioral risk factors for preterm birth include low maternal prepregnancy weight, smoking, substance abuse, and short interpregnancy interval. Only three of our patients had a BMI less than 19; all three of these women had full term deliveries. Additionally, our maternal tobacco use rate was only 6.4%, compared to national averages closer to 13% [77]. All of these factors may have contributed to our low rate of preterm delivery compared to nationally reported averages.

## Conclusion

Vitamin D deficiency was not associated with the composite clinical outcomes of preeclampsia, preterm delivery, intrauterine growth restriction, gestational diabetes, or spontaneous abortion in this study population of nulliparous military members and their beneficiaries. This study was not powered to detect associations between vitamin D status and these individual complications of pregnancy. However, the high percentage of affected individuals highlights the prevalence of vitamin D deficiency and insufficiency in young, healthy reproductive-aged women in our study population. Vitamin D deficiency is a growing topic of interest around the world. Although our study did not demonstrate an association of vitamin D plasma concentration with select clinical outcomes, the prevalence of sub-optimal vitamin D concentrations in patient plasma emphasizes the need for continued research on hypovitaminosis D in pregnancy and reproductive health.

## References

[1] Looker AC, Pfeiffer CM, Lacher DA, Schleicher RL, Picciano MF, Yetley EA. Serum 25-hydroxyvitamin D status of the US population: 1988–1994 compared with 2000–2004. Am J Clin Nutr 2008;88(6):1519–1527 10.3945/ajcn.2008.26182 19064511 PMC2745830

[2] American College of Obstetrics and Gynecology. ACOG committee: Opinion number 495: Vitamin D: screening and supplementation during pregnancy. Obstet Gynecol 2011;118(1):197–198 10.1097/AOG.0b013e318227f06b 21691184

[3] Yetley EA. Assessing the vitamin D status of the US population. Am J Clin Nutr 2008;88(2):558S–564S 18689402 10.1093/ajcn/88.2.558S

[4] Pawley N, Bishop NJ. Prenatal and infant predictors of bone health: the influence of vitamin D. Am J Clin Nutr 2004;80(6 Suppl):1748S–1751S 15585799 10.1093/ajcn/80.6.1748S

[5] Bodnar LM, Simhan HN. Vitamin D may be a link to black-white disparities in adverse birth outcomes. Obstet Gynecol Surv 2010;65(4):273–284 10.1097/OGX.0b013e3181dbc55b 20403218 PMC3222336

[6] Clifton-Bligh RJ, McElduff P, McElduff A. Maternal vitamin D deficiency, ethnicity and gestational diabetes. Diabet Med 2008;25(6):678–684 10.1111/j.1464-5491.2008.02422.x 18544105

[7] Merewood A, Mehta SD, Chen TC, Bauchner H, Holick MF. Association between vitamin D deficiency and primary cesarean section. J Clin Endocrinol Metab 2009;94(3):940–945 10.1210/jc.2008-1217 19106272 PMC2681281

[8] Robinson CJ, Wagner CL, Hollis BW, Baatz JE, Johnson DD. Maternal vitamin D and fetal growth in early-onset severe preeclampsia. Am J Obstet Gynecol 2011;204(6):556 e551–554 10.1016/j.ajog.2011.03.022 21507371 PMC3136573

[9] Zhang C, Qiu C, Hu FB. Maternal plasma 25-hydroxyvitamin D concentrations and the risk for gestational diabetes mellitus. PLoS One 2008;3(11):e3753 10.1371/journal.pone.0003753 19015731 PMC2582131

[10] Bodnar LM, Catov JM, Zmuda JM, Cooper ME, Parrott MS, Roberts JM, et al Maternal serum 25-hydroxyvitamin D concentrations are associated with small-for-gestational age births in white women. J Nutr 2010;140(5):999–1006 10.3945/jn.109.119636 20200114 PMC2855265

[11] Farrant HJ, Krishnaveni GV, Hill JC, Boucher BJ, Fisher DJ, Noonan K, et al Vitamin D insufficiency is common in Indian mothers but is not associated with gestational diabetes or variation in newborn size. Eur J Clin Nutr 2009;63(5):646–652 10.1038/ejcn.2008.14 18285809 PMC2678985

[12] Powe CE, Seely EW, Rana S. First trimester vitamin D, vitamin D binding protein, and subsequent preeclampsia. Hypertension 2010;56(4):758–763 10.1161/HYPERTENSIONAHA.110.158238 20733087 PMC3775612

[13] Christesen HT, Falkenberg T, Lamont RF, Jorgensen JS. The impact of vitamin D on pregnancy: a systematic review. Acta Obstet Gynecol Scand 2012;91(12):1357–1367 10.1111/aogs.12000 22974137

[14] Gestational diabetes mellitus. Practice Bulletin No. 137. American College of Obstetricians and Gynecologists. Obstet Gynecol 2013; 122:406–16. 10.1097/01.AOG.0000433006.09219.f1 23969827

[15] Hypertension in pregnancy. Report of the American College of Obstetricians and Gynecologists’ Task Force on Hypertension in Pregnancy. Obstet Gynecol. 2013 11;122(5):1122–31. 10.1097/01.AOG.0000437382.03963.88 24150027

[16] Management of recurrent pregnancy loss. Practice Bulletin No. 124. American College of Obstetricians and Gynecologists. Int J Gynaecol Obstet 2002;78(2):179–190 12360906 10.1016/s0020-7292(02)00197-2

[17] Management of Stillbirth. ACOG Practice Bulletin No. 102. American College of Obstetricians and Gynecologists. Obstet Gynecol 2009; 113:748–61. 10.1097/AOG.0b013e31819e9ee2 19300347

[18] Ross AC, Taylor CL, Yaktine AL, Del Valle HB. Dietary Reference Intakes for Calcium and Vitamin D. 2011/08/06 ed: National Academies Press (US); 2011 21796828

[19] Holick MF, Binkley NC, Bischoff-Ferrari HA, Gordon CM, Hanley DA, Heaney RP, et al Evaluation, treatment, and prevention of vitamin D deficiency: an Endocrine Society clinical practice guideline. J Clin Endocrinol Metab 2011;96(7):1911–1930 10.1210/jc.2011-0385 21646368

[20] Dolea C, AbouZahr C. Global burden of hypertensive disorders of pregnancy in the year 2000 In: Evidence and Information for Policy (EIP) of the World Health Organization ed; 2003:1–11

[21] Ness RB, Roberts JM. Heterogeneous causes constituting the single syndrome of preeclampsia: a hypothesis and its implications. Am J Obstet Gynecol 1996;175(5):1365–1370 8942516 10.1016/s0002-9378(96)70056-x

[22] Ananth CV, Keyes KM, Wapner RJ. Preeclampsia rates in the United States, 1980–2010: age-period-cohort analysis. BMJ. 2013 11 7; 347:f6564 10.1136/bmj.f6564 24201165 PMC3898425

[23] Roberts CL, Ford JB, Algert CS. Population-based trends in pregnancy hypertension and pre-eclampsia: an international comparative study. BMJ Open 2011;1(1):e000101 10.1136/bmjopen-2011-000101 22021762 PMC3191437

[24] Samadi AR, Mayberry RM, Zaidi AA, Pleasant JC, McGhee N Jr, Rice RJ. Maternal hypertension and associated pregnancy complications among African-American and other women in the United States. Obstet Gynecol 1996;87(4):557–563 8602308 10.1016/0029-7844(95)00480-7

[25] Sibai BM, Caritis S, Hauth J. What we have learned about preeclampsia. Semin Perinatol 2003;27(3):239–246 12889591 10.1016/s0146-0005(03)00022-3

[26] Wallis AB, Saftlas AF, Hsia J, Atrash HK. Secular trends in the rates of preeclampsia, eclampsia, and gestational hypertension, United States, 1987–2004. Am J Hypertens 2008;21(5):521–526 10.1038/ajh.2008.20 18437143

[27] Martin JA, Hamilton BE, Osterman MJ. Births in the United States, 2013. NCHS Data Brief. 2014 12;(175):1–8. 25483923

[28] Beck S, Wojdyla D, Say L. The worldwide incidence of preterm birth: a systematic review of maternal mortality and morbidity. Bull World Health Organ 2010;88(1):31–38 10.2471/BLT.08.062554 20428351 PMC2802437

[29] Lifshitz F, Grimberg A. Growth and Growth Disorders: Worrisome Growth In: Lifshitz F ed, Pediatric Endocrinology. 5 ed. New York: Informa Healthcare USA, Inc.; 2007:1–50

[30] Romo A, Carceller R, Tobajas J. Intrauterine growth retardation (IUGR): epidemiology and etiology. Pediatr Endocrinol Rev 2009;6 Suppl 3:332–336 19404231

[31] Albrecht SS, Kuklina EV, Bansil P, Jamieson DJ, Whiteman MK, Kourtis AP, et al Diabetes trends among delivery hospitalizations in the U.S., 1994–2004. Diabetes Care 2010;33(4):768–773 10.2337/dc09-1801 20067968 PMC2845025

[32] Anna V, van der Ploeg HP, Cheung NW, Huxley RR, Bauman AE. Sociodemographic correlates of the increasing trend in prevalence of gestational diabetes mellitus in a large population of women between 1995 and 2005. Diabetes Care 2008;31(12):2288–2293 10.2337/dc08-1038 18809630 PMC2584183

[33] DeSisto CL, Kim SY, Sharma AJ. Prevalence Estimates of Gestational Diabetes Mellitus in the United States, Pregnancy Risk Assessment Monitoring System (PRAMS), 2007–2010. Prev Chronic Dis 2014;11:130415. 10.5888/pcd11.130415 PMC4068111 24945238

[34] Dabelea D, Snell-Bergeon JK, Hartsfield CL, et al Increasing prevalence of gestational diabetes mellitus (GDM) over time and by birth cohort: Kaiser Permanente of Colorado GDM Screening Program. Diabetes Care 2005;28(3):579–584 15735191 10.2337/diacare.28.3.579

[35] Getahun D, Nath C, Ananth CV, Chavez MR, Smulian JC. Gestational diabetes in the United States: temporal trends 1989 through 2004. Am J Obstet Gynecol 2008;198(5):525 e521–525 10.1016/j.ajog.2007.11.017 18279822

[36] Guariguata L, Linnenkamp U, Beagley J, Whiting DR, Cho NH. Global estimates of the prevalence of hyperglycaemia in pregnancy. Diabetes Res Clin Pract. 2014 2;103(2):176–85. 10.1016/j.diabres.2013.11.003 24300020

[37] Wang X, Chen C, Wang L. Conception, early pregnancy loss, and time to clinical pregnancy: a population-based prospective study. Fertil Steril 2003;79(3):577–584 12620443 10.1016/s0015-0282(02)04694-0

[38] Wilcox AJ, Baird DD, Weinberg CR. Time of implantation of the conceptus and loss of pregnancy. N Engl J Med 1999;340(23):1796–1799 10362823 10.1056/NEJM199906103402304

[39] Wei SQ, Qi HP, Luo ZC, Fraser WD. Maternal Vitamin D Status and Adverse Pregnancy Outcomes: A Systematic Review and Meta-Analysis. J Matern Fetal Neonatal Med 2013 10.3109/14767058.2013.765849 23311886

[40] Johnson DD, Wagner CL, Hulsey TC, McNeil RB, Ebeling M, Hollis BW. Vitamin D deficiency and insufficiency is common during pregnancy. Am J Perinatol 2011;28(1):7–12 10.1055/s-0030-1262505 20640974

[41] Thomas MK, Lloyd-Jones DM, Thadhani RI. Hypovitaminosis D in medical inpatients. N Engl J Med 1998;338(12):777–783 9504937 10.1056/NEJM199803193381201

[42] Ginde AA, Sullivan AF, Mansbach JM, Camargo CA Jr. Vitamin D insufficiency in pregnant and nonpregnant women of childbearing age in the United States. Am J Obstet Gynecol 2010;202(5):436 e431–438 10.1016/j.ajog.2009.11.036 20060512 PMC3784988

[43] Hollis BW, Wagner CL. Vitamin d and pregnancy: skeletal effects, nonskeletal effects, and birth outcomes. Calcif Tissue Int 2013;92(2):128–139 10.1007/s00223-012-9607-4 22623177

[44] Wagner CL, McNeil R, Hamilton SA, Winkler J, Rodriguez Cook C, Warner G. A randomized trial of vitamin D supplementation in 2 community health center networks in South Carolina. Am J Obstet Gynecol 2012 10.1016/j.ajog.2012.10.888 PMC4365423 23131462

[45] Rosen CJ. Clinical practice. Vitamin D insufficiency. N Engl J Med 2011;364(3):248–254 10.1056/NEJMcp1009570 21247315

[46] McCullough ML, Bostick RM, Daniel CR. Vitamin D status and impact of vitamin D3 and/or calcium supplementation in a randomized pilot study in the Southeastern United States. J Am Coll Nutr 2009;28(6):678–686 20516268 10.1080/07315724.2009.10719801 PMC3731379

[47] Webb AR, Kline L, Holick MF. Influence of season and latitude on the cutaneous synthesis of vitamin D3: exposure to winter sunlight in Boston and Edmonton will not promote vitamin D3 synthesis in human skin. J Clin Endocrinol Metab 1988;67(2):373–378 2839537 10.1210/jcem-67-2-373

[48] Climatemps.com http://www.seattle.climatemps.com/ Accessed January 5, 2015.

[49] Climatemps.com http://www.columbia-sc.climatemps.com/ Accessed January 5, 2015

[50] Azar M, Basu A, Jenkins AJ. Serum carotenoids and fat-soluble vitamins in women with type 1 diabetes and preeclampsia: a longitudinal study. Diabetes Care 2011;34(6):1258–1264 10.2337/dc10-2145 21498785 PMC3114346

[51] Baker AM, Haeri S, Camargo CA Jr, Stuebe AM, Boggess KA. A nested case-control study of first-trimester maternal vitamin D status and risk for spontaneous preterm birth. Am J Perinatol 2011;28(9):667–672 10.1055/s-0031-1276731 21500145 PMC4372898

[52] Baker AM, Haeri S, Camargo CA Jr, Stuebe AM, Boggess KA. First-trimester maternal vitamin D status and risk for gestational diabetes (GDM) a nested case-control study. Diabetes Metab Res Rev 2012;28(2):164–168 10.1002/dmrr.1282 21818838 PMC4381548

[53] Bodnar LM, Catov JM, Simhan HN, Holick MF, Powers RW, Roberts JM. Maternal vitamin D deficiency increases the risk of preeclampsia. J Clin Endocrinol Metab 2007;92(9):3517–3522 17535985 10.1210/jc.2007-0718 PMC4288954

[54] Brooke OG, Brown IR, Bone CD. Vitamin D supplements in pregnant Asian women: effects on calcium status and fetal growth. Br Med J 1980;280(6216):751–754 6989438 10.1136/bmj.280.6216.751 PMC1600591

[55] Brough L, Rees GA, Crawford MA, Morton RH, Dorman EK. Effect of multiple-micronutrient supplementation on maternal nutrient status, infant birth weight and gestational age at birth in a low-income, multi-ethnic population. Br J Nutr 2010;104(3):437–445 10.1017/S0007114510000747 20412605

[56] Burris HH, Rifas-Shiman SL, Camargo CA Jr. Plasma 25-hydroxyvitamin D during pregnancy and small-for-gestational age in black and white infants. Ann Epidemiol 2012;22(8):581–586 10.1016/j.annepidem.2012.04.015 22658824 PMC3396717

[57] Burris HH, Rifas-Shiman SL, Kleinman K, Litonjua AA, Huh SY, Rich-Edwards JW. Vitamin D deficiency in pregnancy and gestational diabetes mellitus. Am J Obstet Gynecol 2012;207(3):182 e181–188 10.1016/j.ajog.2012.05.022 22717271 PMC3432741

[58] Christian P, West KP, Khatry SK. Effects of maternal micronutrient supplementation on fetal loss and infant mortality: a cluster-randomized trial in Nepal. Am J Clin Nutr 2003;78(6):1194–1202 14668283 10.1093/ajcn/78.6.1194

[59] Delvin EE, Salle BL, Glorieux FH, Adeleine P, David LS. Vitamin D supplementation during pregnancy: effect on neonatal calcium homeostasis. J Pediatr 1986;109(2):328–334 3488384 10.1016/s0022-3476(86)80396-1

[60] Dunlop AL, Taylor RN, Tangpricha V, Fortunato S, Menon R. Maternal micronutrient status and preterm versus term birth for black and white US women. Reprod Sci 2012;19(9):939–948 10.1177/1933719112438442 22527984 PMC4046315

[61] Fernandez-Alonso AM, Dionis-Sanchez EC, Chedraui P, Gonzalez-Salmeron MD, Perez-Lopez FR. First-trimester maternal serum 25-hydroxyvitamin D(3) status and pregnancy outcome. Int J Gynaecol Obstet 2012;116(1):6–9 10.1016/j.ijgo.2011.07.029 21959069

[62] Gernand AD, Simhan HN, Klebanoff MA, Bodnar LM. Maternal serum 25-hydroxyvitamin d and measures of newborn and placental weight in a u.s. Multicenter cohort study. J Clin Endocrinol Metab 2013;98(1):398–404 10.1210/jc.2012-3275 23162094 PMC3537090

[63] Halhali A, Tovar AR, Torres N, Bourges H, Garabedian M, Larrea F. Preeclampsia is associated with low circulating levels of insulin-like growth factor I and 1,25-dihydroxyvitamin D in maternal and umbilical cord compartments. J Clin Endocrinol Metab 2000;85(5):1828–1833 10843160 10.1210/jcem.85.5.6528

[64] Haugen M, Brantsaeter AL, Trogstad L. Vitamin D supplementation and reduced risk of preeclampsia in nulliparous women. Epidemiology 2009;20(5):720–726 10.1097/EDE.0b013e3181a70f08 19451820

[65] Hollis BW, Johnson D, Hulsey TC, Ebeling M, Wagner CL. Vitamin D supplementation during pregnancy: double-blind, randomized clinical trial of safety and effectiveness. J Bone Miner Res 2011;26(10):2341–2357 10.1002/jbmr.463 21706518 PMC3183324

[66] Hypponen E, Hartikainen AL, Sovio U, Jarvelin MR, Pouta A. Does vitamin D supplementation in infancy reduce the risk of pre-eclampsia? Eur J Clin Nutr 2007;61(9):1136–1139 17268418 10.1038/sj.ejcn.1602625

[67] Kolusari A, Kurdoglu M, Yildizhan R. Catalase activity, serum trace element and heavy metal concentrations, and vitamin A, D and E levels in pre-eclampsia. J Int Med Res 2008;36(6):1335–1341 19094444 10.1177/147323000803600622

[68] Leffelaar ER, Vrijkotte TG, van Eijsden M. Maternal early pregnancy vitamin D status in relation to fetal and neonatal growth: results of the multi-ethnic Amsterdam Born Children and their Development cohort. Br J Nutr 2010;104(1):108–117 10.1017/S000711451000022X 20193097

[69] Maghbooli Z, Hossein-Nezhad A, Karimi F, Shafaei AR, Larijani B. Correlation between vitamin D3 deficiency and insulin resistance in pregnancy. Diabetes Metab Res Rev 2008;24(1):27–32 17607661 10.1002/dmrr.737

[70] Makgoba M, Nelson SM, Savvidou M, Messow CM, Nicolaides K, Sattar N. First-trimester circulating 25-hydroxyvitamin D levels and development of gestational diabetes mellitus. Diabetes Care 2011;34(5):1091–1093 10.2337/dc10-2264 21454797 PMC3114479

[71] Labriji-Mestaghanmi H, Billaudel B, Garnier PE, Malaisse WJ, Sutter BC. Vitamin D and pancreatic islet function. I. Time course for changes in insulin secretion and content during vitamin D deprivation and repletion. J Endocrinol Invest 1988;11(8):577–584 3072373 10.1007/BF03350185

[72] Burris HH, Camargo CA Jr. Vitamin D and gestational diabetes mellitus. Curr Diab Rep. 2014 1;14(1):451 10.1007/s11892-013-0451-3 24277676 PMC3895371

[73] Katon J, Mattocks K, Zephyrin L, Reiber G, Yano EM, Callegari L, et al Gestational diabetes and hypertensive disorders of pregnancy among women veterans deployed in service of operations in Afghanistan and Iraq. J Womens Health (Larchmt). 2014 10;23(10):792–800. 10.1089/jwh.2013.4681 25090022 PMC4195229

[74] Abouzeid M, Versace VL, Janus ED, Davey MA, Philpot B, Oats J, et al A population-based observational study of diabetes during pregnancy in Victoria, Australia, 1999–2008. BMJ Open. 2014 11 14;4(11):e005394 10.1136/bmjopen-2014-005394 25398676 PMC4244457

[75] Hossein-Nezhad A, Holick MF. Vitamin D for health: a global perspective. Mayo Clin Proc. 2013 7;88(7):720–55. 10.1016/j.mayocp.2013.05.011 23790560 PMC3761874

[76] Prediction and prevention of preterm birth. Practice Bulletin No. 130. American College of Obstetricians and Gynecologists. Obstet Gynecol 2012;120:964–73. 22996126 10.1097/AOG.0b013e3182723b1b

[77] Smoking cessation during pregnancy. Committee Opinion No. 471. American College of Obstetricians and Gynecologists. Obstet Gynecol 2010;116:1241–4. 10.1097/AOG.0b013e3182004fcd 20966731

